# Free-Breathing StarVIBE Sequence for the Detection of Extranodal Extension in Head and Neck Cancer: An Image Quality and Diagnostic Performance Study

**DOI:** 10.3390/cancers15204992

**Published:** 2023-10-15

**Authors:** Jiangming Qu, Tong Su, Boju Pan, Tao Zhang, Xingming Chen, Xiaoli Zhu, Yu Chen, Zhuhua Zhang, Zhengyu Jin

**Affiliations:** 1 Department of Radiology, Peking Union Medical College Hospital, Chinese Academy of Medical Sciences, No.1 Shuai Fu Yuan, Dong Cheng District, Beijing 100730, China; 2 Department of Pathology, Peking Union Medical College Hospital, Chinese Academy of Medical Sciences, No.1 Shuai Fu Yuan, Dong Cheng District, Beijing 100730, China; 3 Department of Stomatology, Peking Union Medical College Hospital, Chinese Academy of Medical Sciences, No.1 Shuai Fu Yuan, Dong Cheng District, Beijing 100730, China; 4 Department of Otolaryngology, Peking Union Medical College Hospital, Chinese Academy of Medical Sciences, No.1 Shuai Fu Yuan, Dong Cheng District, Beijing 100730, China

**Keywords:** extranodal extension, magnetic resonance imaging, free-breathing, head and neck cancer

## Abstract

**Simple Summary:**

Extranodal extension (ENE) represents a critical pathologic high-risk factor for disease progression in head and neck cancer. Precise pre-treatment imaging to detect the presence or absence of ENE could facilitate the selection of appropriate initial therapy. Previous studies utilizing CT or MRI for detecting ENE have shown high specificity but modest sensitivity. This study demonstrates improved image quality of cervical lymph nodes using a free-breathing MRI sequence (StarVIBE), which is highly resistant to respiratory motion. Based on node-to-node matched pathology, a composite diagnostic criterion derived from StarVIBE was proposed to potentially enhance the accurate detection of ENE.

**Abstract:**

(1) Background: This study aims to evaluate the image quality of abnormal cervical lymph nodes in head and neck cancer and the diagnostic performance of detecting extranodal extension (ENE) using free-breathing StarVIBE. (2) Methods: In this retrospective analysis, 80 consecutive head and neck cancer patients underwent StarVIBE before neck dissection at an academic center. Image quality was compared with conventional VIBE available for 28 of these patients. A total of 73 suspicious metastatic lymph nodes from 40 patients were found based on morphology and enhancement pattern on StarVIBE. Sensitivity (SN), specificity (SP), and odds ratios were calculated for each MR feature from StarVIBE to predict pathologic ENE. (3) Results: StarVIBE showed significantly superior image quality, signal-to-noise ratio (SNR), and contrast-to-noise ratio (CNR) for enlarged lymph nodes compared to VIBE. The MR findings of “invading adjacent planes” (SN, 0.54; SP, 1.00) and “matted nodes” (SN, 0.72; SP, 0.89) emerged as notable observations. The highest diagnostic performance was attained by combining these two features (SN, 0.93; SP, 0.89). (4) Conclusions: This study confirms that StarVIBE offers superior image quality for abnormal lymph nodes compared to VIBE, and it can accurately diagnose ENE by utilizing a composite MR criterion in head and neck cancer.

## 1. Introduction

Extranodal extension (ENE) is defined as the spread of tumor cells beyond the capsule of a metastatic lymph node into the perinodal tissues and reflects the aggressiveness of a tumor. In patients with head and neck cancer, ENE is significantly associated with increased rates of locoregional recurrence, distant metastasis, and poorer overall survival, making it a crucial poor prognostic factor [1,2,3,4,5]. The gold standard for the diagnosis of ENE requires histopathological assessment of removed lymph nodes and is determined only when patients undergo neck dissection. However, the use of imaging-based pre-treatment identification of ENE has the potential to alter this scenario by enabling the planning of optimal treatment options before initial intervention [6,7,8].

CT and MRI demonstrate comparable diagnostic performance in detecting ENE, as indicated by recent meta-analyses that found no significant differences in pooled sensitivity and specificity between the two modalities [9,10]. The advantage of MRI over CT is the higher soft tissue contrast; however, due to its longer acquisition time, MRI is more susceptible to breathing, swallowing, and other sources of movement artifacts, and therefore, MRI is limited in its ability to evaluate ENE [11]. Currently, a small number of studies have reported the use of MRI in the diagnosis of ENE, with high specificity but merely modest sensitivity [12,13,14,15,16].

Gradient echo (GRE) sequence with the volumetric interpolated breath-hold examination (VIBE) provides high-resolution 3D imaging but requires breath-hold for better image quality [17], which might be difficult for head and neck cancer patients if the tumor blocks the upper aerodigestive tract. More recently, an innovative 3D-T1-GRE Stack-of-Stars VIBE (StarVIBE) sequence permitted robust free-breathing examinations. It employs the radial center overlap filling method based on radial acquisition in the k-space of gradient echoes to effectively reduce motion artifacts and provide excellent resolution [18,19]. Previous studies have reported that the application of StarVIBE can successfully improve the evaluation of thoracic and abdominal lesions and is especially suitable for pediatric patients and fetuses [19,20,21,22,23]. However, there are no existing studies examining the use of StarVIBE in assessing neck lesions. Our hypothesis posits that StarVIBE could offer superior image quality and thus help to evaluate ENE in head and neck cancer.

Therefore, this study aimed to compare the image quality of abnormal lymph nodes between StarVIBE and conventional VIBE in head and neck cancer. We also performed a node-based analysis to examine the diagnostic performance of several MR features associated with ENE using the StarVIBE sequence compared with pathology.

## 2. Materials and Methods

### 2.1. Patient Selection

**Figure 1:**
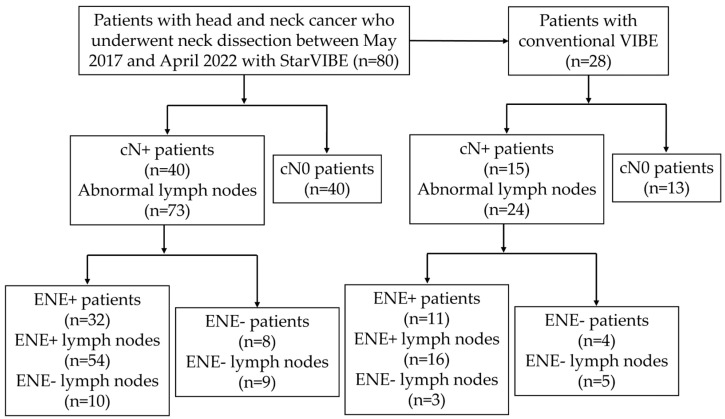
Derivation of study population and analyses schema.

This study was approved by the institutional review board. Eighty consecutive patients with locally advanced head and neck cancer were referred to the Department of Radiology, Peking Union Medical College Hospital, for preoperative contrast-enhanced neck soft tissue MRI, including StarVIBE, before selective neck dissection between May 2017 and March 2022. Clinical data were collected from institutional electronic medical records. Twenty-eight individuals who underwent the examination prior to February 2019 also had a VIBE sequence that was routinely used in clinical practice. The patients were categorized for ENE evaluation as cN0 if their preoperative StarVIBE scans did not reveal any abnormal lymph nodes or as cN+ if their preoperative StarVIBE indicated abnormal lymph nodes (Figure 1). The criteria for identifying abnormal lymph nodes on StarVIBE and VIBE were as follows: (1) Morphology: lymph nodes without a hilum and larger than 5 mm. (2) Enhancement: abnormally inhomogeneous enhancement.

### 2.2. MRI Acquisition

The examinations were performed on a clinical 3-Tesla scanner (MAGNETOM Skyra, Siemens Healthcare, Erlangen, Germany) equipped with a 20-channel head–neck coil. After administration of gadoteridol contrast agent at a weight-adjusted dose of 0.1 mmol/kg at a flow rate of 2 mL/s, dynamic contrast-enhanced MRI (DCE-MRI) was initially acquired, followed by StarVIBE and then conventional VIBE in a fixed sequential order. StarVIBE was acquired using the following parameters: orientation: 3D; repetition time (TR): 4.5 ms; echo time (TE): 1.6 ms; slice thickness: 1 mm; matrix size: 224 × 224; field of view (FOV): 224 × 224 mm^2^; bandwidth: 770.0 Hz/pixel; acquisition time (TA): 228 s. VIBE was acquired using the following parameters: orientation: transverse; TR: 6.7 ms; TE: 2.5 ms; slice thickness: 1 mm; matrix size: 256 × 256; FOV: 260 × 260 mm^2^; bandwidth: 650.0 Hz/pixel; TA: 137 s. Controlled aliasing in parallel imaging results in higher acceleration (CAIPIRINHA) applied to VIBE, with an acceleration factor of 2. Four image sets of VIBE were obtained via the two-point Dixon method: an in-phase image, an opposed-phase image, a fat-only image, and a water-only image, with the water-reconstruction image being compared to the StarVIBE sequence with fast fat saturation.

### 2.3. Radiological Assessment

Two head and neck radiologists (Y.C., 15 years experience; T.S., 6 years experience) analyzed MR images independently, blinded to the pathological findings. For the statistical analysis, consensus conclusions were used. Qualitative evaluation was assessed by the image quality of lymph nodes graded on a 5-point scale (1 = worse: contour not identifiable; 2 = poor: nodes identifiable but heavily mixed with the background; 3 = acceptable: contour moderately mixed with the background; 4 = good: well-defined contour and good contrast with the background; and 5 = excellent: perfectly defined contour and high contrast with surrounding structures) and by motion and aliasing artifacts graded on a 5-point scale (1, unreadable; 2, extreme artifacts; 3, moderate artifacts; 4, mild artifacts; 5, none). Quantitative assessment was performed using the signal-to-noise ratio (SNR) and the contrast-to-noise ratio (CNR). Regions of interest (ROI) of target tissue were manually drawn on StarVIBE or VIBE images in the same axial plane. The necrotic area was avoided in the lymph node ROI. The following formulas define the SNR and the CNR:

$$\mathrm{SNR}=\frac{{\mathrm{SI}}_{\mathrm{nodes}}}{{\mathrm{SD}}_{\mathrm{nodes}}}$$

$$\mathrm{CNR}=\frac{\left|{\mathrm{SI}}_{\mathrm{nodes}}-{\mathrm{SI}}_{\mathrm{muscles}}\right|}{\sqrt{{\mathrm{SD}}_{\mathrm{nodes}}{}^{2}+{\mathrm{SD}}_{\mathrm{muscles}}{}^{2}}}$$

Four MR features of ENE are defined as follows: (1) Irregular nodal margin: lymph node with indistinct or irregular margins. (2) Infiltrating adjacent planes: abnormal enhancement in planes adjacent to the lymph node. (3) Matted nodes: a conglomerate of 2 or more abnormal lymph nodes with an absence of internodal fat planes. (4) Nodal necrosis: lymph node with the non-enhancing area. The long axial diameter, defined as the longest diameter of the lymph node in any plane, and the short axial diameter, defined as the maximum diameter of the lymph node perpendicular to its long axis, were recorded. If the nodes were matted into a larger lesion, the diameter of the whole lesion was measured.

### 2.4. Matching to Pathologic Examination

The surgeon in the operating room labeled all neck dissection specimens according to the neck levels. The pathologist manually identified and localized the lymph nodes within each neck level in the specimen. Each lymph node was examined microscopically to determine the presence or absence of tumor cells, and the maximum diameter of metastatic lymph nodes was recorded. Subsequently, the presence or absence of ENE was examined in each metastatic lymph node. Pathologic ENE is defined as tumor cell growth beyond the capsule of a metastatic lymph node. Additionally, deposits of carcinoma in soft tissues that replace the lymph node entirely are also included in ENE. However, it is not considered ENE when tumor cells grow into the surrounding capsule but not through or beyond it. The matching of metastatic lymph nodes on MRI and surgical specimen pathology was determined based on the neck levels and the largest diameter of the lymph nodes.

### 2.5. Statistical Analysis

Statistical analyses were performed by using Statistical Package for the Social Sciences (SPSS, version 26.0, IBM Corp., Armonk, NY, USA) and MedCalc Statistical Software (version 22.009, MedCalc Software Ltd., Ostend, Belgium). Two-sided *p* < 0.05 was considered significant. Data are medians with interquartile ranges in parenthesis or means ± standard deviations. The paired-sample *t*-test or the Mann–Whitney *U* test was used to compare normally or non-normally distributed data. The sensitivity, specificity, and accuracy of MR features of ENE were calculated using pathological results as the gold standard. The 95% confidence intervals for sensitivity, specificity, and accuracy were “exact” Clopper–Pearson confidence intervals. Inter-rater agreement was assessed by estimating the intraclass correlation coefficients (ICCs) and interpreted as follows: poor inter-rater agreement < 0.40; fair = 0.40–0.59; good = 0.60–0.74; and excellent = 0.75–1.00. The Youden index method was adopted to determine the optimal cut-off value for continuous variables. Binary logistic regression was used to examine the association between variables and ENE. The combination of two or more features refers to at least one feature.

## 3. Results

### 3.1. Patient Characteristics

The characteristics and demographics of the cohort are summarized in Table 1. Of the 80 patients who underwent StarVIBE before neck dissection, 73 radiologically abnormal lymph nodes were found in 40 patients (cN+). The remaining 40 cN0 patients underwent prophylactic neck dissection due to clinical high-risk factors. Of the 40 cN+ patients, 32 underwent primary surgery for neck dissection and primary tumor excision, five underwent salvage surgery for nodal recurrence, and three underwent salvage surgery for resistance to induction chemoimmunotherapy. Of the 40 patients whose StarVIBE was negative for any metastatic lymph nodes, three were later found to have occult lymph node metastasis on pathologic examination, and two of these three patients had occult ENE. Imaging to surgery interval was less than four weeks.

### 3.2. Comparison between StarVIBE and VIBE

In the study, out of 28 patients with available VIBE for comparison, twenty-four abnormal lymph nodes were identified from 15 patients for image quality analysis. Seven patients (47%) had been diagnosed with oral cavity cancer, one patient (7%) had laryngeal cancer, six patients (40%) had hypopharyngeal cancer, and one patient (7%) had salivary gland cancer. Six nodes (25%) were located in Level I, seven nodes (29%) were in Level II, five nodes (21%) were in Level III, and six nodes (25%) were in Level IV. The average number of abnormal lymph nodes per patient was 1.6 (range 1–3; SD 0.5). StarVIBE delivered a significantly superior image quality compared to VIBE, considering the overall image quality, motion artifacts, aliasing artifacts, SNR, and CNR (Table 2). No significant difference in image quality was detected across lymph nodes at different neck levels. The inter-rater agreement on image quality ranged from fair to excellent (ICC 0.51–0.92; Table 2).

### 3.3. Association of MR Features with Histologically Confirmed ENE

Among cN+ patients, there were no significant differences in age, gender, primary tumor site, and T staging. However, ENE tended to occur in metastatic carcinoma from the hypopharynx, supraglottic regions, and submandibular glands (Table 3). Sixteen nodes (22%) were located in Level I, 20 nodes (27%) were found in Level II, 18 nodes (25%) were located in Level III, 11 nodes (15%) were found in Level IV, 7 nodes in level V (9.6%), and 1 node in level VI (0.2%). Subcentimeter lymph nodes, defined as <1 cm in the short axis, accounted for 43.8% (*n* = 32) of the total lymph nodes examined. The mean number of positive nodes was 1.8 (range 1–7; SD 1.2) per patient. Histopathological examination revealed that 95.9% (*n* = 70) of the 73 abnormal lymph nodes were metastatic, while the remaining 4.1% (*n* = 3) showed signs of acute or chronic inflammation. Of the abnormal nodes, 54 were positive for ENE, while 19 were negative.

**Figure 2:**
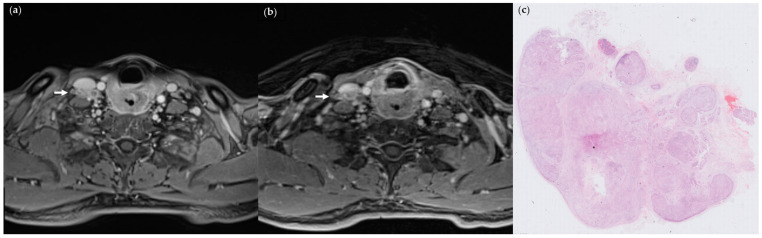
(**a**) StarVIBE image of a 46-year-old male patient with pT4aN3bM0 hypopharyngeal cancer invading esophageal muscle, showing level IV matted nodes on the right side (arrows); (**b**) VIBE image of the same section; (**c**) pathologic section through the matted node, showing ENE. (Hematoxylin-eosin stain; 4×).

**Figure 3:**
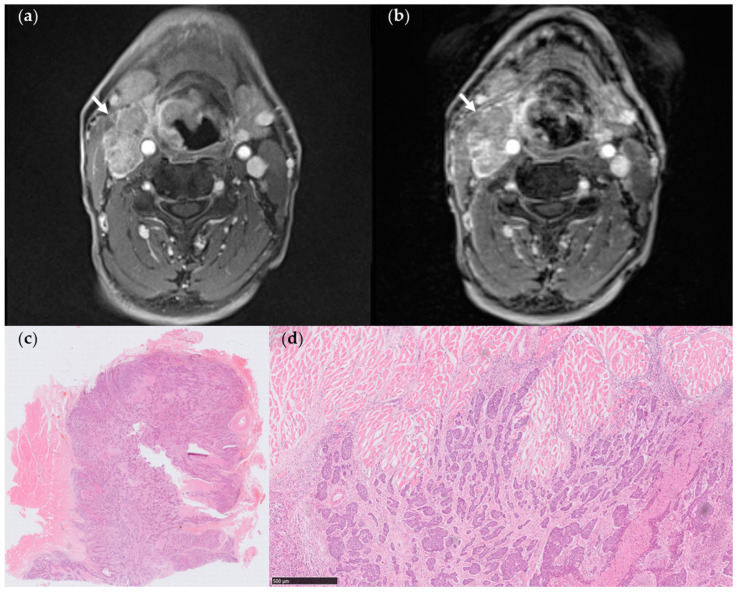
(**a**) StarVIBE image of a 60-year-old male patient with pT3N3bM0 hypopharyngeal cancer. The level III matted nodes on the right side showed a distinct margin with the sternocleidomastoid muscle; however, there was a suspicious invasion of the adjacent muscle (arrows). (**b**) VIBE image of the same section revealed level III matted nodes on the right side with a less distinct margin between the lymph nodes and adjacent muscle (arrows) attributed to low CNR. (**c**,**d**) Pathologic section through one of the matted nodes. The metastatic lymph node was in close proximity to the striated muscle, showing only a local extension into the adjacent muscle (arrows), while the remaining margin appeared to be absent of ENE (Hematoxylin-eosin stain, (**c**), 4×, (**d**), 50×).

Following the matching of abnormal lymph nodes with metastases on StarVIBE to the pathology results, the sensitivities, specificities, and accuracies of each feature were presented in Table 4. The inter-rater agreement was excellent: invading adjacent planes (ICC, 0.97 [0.96–0.98]), nodal necrosis (ICC, 0.88 [0.82–0.92]), matted nodes (ICC, 0.95 [0.92–0.97]), long-axis diameter (ICC, 0.99 [0.99–1.00]), except for irregular nodal margins (ICC, 0.56 [0.38–0.70]). The long-axis diameter of ENE+ lymph nodes was significantly greater compared to those without ENE (11.3 ± 2.6 mm vs. 21.1 ± 10.4 mm, *p* < 0.001), with a cut-off value of 14.8 mm. Both nodal necrosis and long-axis diameter had the highest sensitivity (SN 0.74), while the sensitivities of the other MR features ranged from 0.26 to 0.72. Infiltrating adjacent planes showed the highest specificity (SP 1.00), whereas the specificities of the other features ranged from 0.73 to 0.89. Irregular nodal margins were infrequent in StarVIBE images (SN = 0.26, SP = 0.74). In univariate analysis, nodal necrosis, matted nodes (Figure 2 and Figure 3), and long axial diameter were found to be significant determinants for pathologic ENE; however, in multivariable analysis, matted nodes had a continuing significant impact on ENE (Table 5). The highest accuracy was achieved by combining the infiltration of adjacent planes and matted nodes as a composite criterion.

Based on the composite criterion, six lymph nodes (8.2%) yielded different diagnostic results when comparing the combined criteria (invading adjacent planes + matted nodes) on StarVIBE and histopathology. Out of the two false-positive nodes indicating matted nodes, one was categorized as having growth into the surrounding capsule but not through or beyond, which does not meet the criteria for pathologic ENE. Four false-negative nodes did not exhibit invasion into adjacent planes nor the presence of matted nodes. Out of the 24 abnormal lymph nodes examined using both conventional VIBE and StarVIBE, 15 out of 16 ENE+ lymph nodes were accurately diagnosed by StarVIBE. Three lymph nodes yielded conflicting diagnoses between VIBE and StarVIBE, with all three being false negatives of invading adjacent planes on VIBE.

## 4. Discussion

This study presents the application of the free-breathing StarVIBE sequence for diagnosing ENE in head and neck cancer for the first time. The results of our study indicate that StarVIBE has the potential to surpass conventional VIBE as a more effective method for assessing ENE, which is supported by the improved SNR, CNR, subjective image quality, and reduction of artifacts achieved with StarVIBE. Furthermore, the in-depth node-based analysis revealed that matted nodes on StarVIBE emerged as a significant independent determinant based on multivariate analysis. By effectively combining the features of invading adjacent planes and matted nodes, StarVIBE demonstrated strong predictive capabilities for detecting pathologic ENE with high sensitivity and specificity.

Currently, there has been no investigation on the use of free-breathing MRIs like StarVIBE for ENE detection. King et al. [12] and Kimura et al. [13] acquired MR images using a conventional contrast-enhanced spin-echo sequence. Frood et al. [14] found, through MR texture analysis of the T1-weighted spin-echo sequence, that combining nodal entropy with irregular contour yielded the most accurate prediction of ENE, and Lodder et al. [15] employed contrast-enhanced 3D THRIVE, a technique akin to VIBE but not free-breathing. Overall, the diagnostic performance of previous MRI studies exhibits low sensitivity but high specificity, resulting in a pooled sensitivity and specificity of 0.60 (0.49–0.70) and 0.96 (0.85–0.99), respectively [24].

StarVIBE potentially surpasses previous MRI studies in diagnosing ENE by providing increased sensitivity without compromising specificity, owing to various possible advantages. Firstly, the free-breathing StarVIBE delineated sharper lymph node boundaries compared to VIBE, which was often compromised by artifacts; this implies that StarVIBE more precisely illustrates the local tumor cell infiltration surrounding the planes of lymph nodes. Secondly, the StarVIBE sequence offered a higher image quality of the fine structure of lymph nodes, and some enlarged lymph nodes on CT could be identified as matted nodes on StarVIBE. Meanwhile, we did not discover the “lobulated contour” sign [15,25] of single lymph nodes on StarVIBE, which suggested that the lobulated contour of lymph nodes might be formed by nodal matting. Lastly, both SNR and CNR were improved on the StarVIBE sequence with excellent contrast-agent sensitivity, enhancing the distinction between lymph nodes and adjacent muscle structures since CNR on the VIBE sequence was close to zero, making VIBE more prone to overly estimate the presence of ENE.

Our study highlights the high sensitivity and specificity of matted nodes. Previously, the presence of matted nodes was not a conventional feature for ENE and was only incorporated in very few CT studies with low sensitivity (ranging from 0.13 to 0.50) but high specificity (ranging from 0.86 to 0.97) [25,26,27,28,29,30]. The prevalence of matted nodes on CT was relatively low, and the primary sites were mainly oropharyngeal cancer. On the contrary, our study attached great importance to matted nodes, demonstrating that nodal matting has the highest sensitivity among all features and was an independent predictor for ENE. Matted nodes can additionally supplement the sign of invading adjacent planes to further increase diagnostic performance.

Similarly, “matted nodes” are a prevalent radiologic feature in nasopharyngeal cancer, serving as an independent prognostic factor to predict distant metastasis-free survival [31,32]. Lu et al. [33] categorized the extent of radiologic extranodal extension (rENE) into three grades for nasopharyngeal cancer: Grade 1 represents infiltration into the surrounding fat, Grade 2 refers to matted nodes, and Grade 3 involves invasion into adjacent structures. In fact, Grade 2 ENE (matted nodes) is prevalent in 51% (487 out of 826 patients) of all rENE patients in nasopharyngeal cancer [33]. Among our cohort, the radiological prevalence of nodal matting is 57.5% (23 out of 40 patients). Furthermore, several studies have previously evaluated the poor prognostic impact of matted nodes on CT or MRI in head and neck patients, but with no available pathologic results to confirm the presence of ENE [34,35,36,37,38,39]. Additional studies are necessary to elucidate the relationship between the formation of matted nodes and ENE. To date, no study has explored matted nodes as predictors of pathologic ENE. We speculate that the higher image quality of StarVIBE for soft tissue may enhance the detection of the internal structure of large lymph nodes and facilitate the identification of additional matted nodes. Lastly, there is a general trend that a larger metastatic node is associated with a higher likelihood of ENE [13,15,28]. In our study, the optimal threshold for the long axial diameter was determined to be 14.8 mm (SN 0.74, SP 0.89), and the presence of matted nodes can also contribute to larger diameters as they are considered one node for measurement purposes.

In previous CT studies, central necrosis exhibited the highest pooled sensitivity (SN 0.81) among other features while maintaining a pooled specificity of 0.65 [9]. The presence of nodal necrosis sign on StarVIBE exhibited compromised sensitivity (SN 0.74) and specificity (SP 0.58). This can be attributed to the fact that ENE occurs at the margin of nodes, while nodal necrosis takes place inside the nodes, which means nodal necrosis is not a direct indicator of ENE. Infiltration of adjacent planes exhibited low sensitivity and high specificity in both previous CT and MRI studies. It is possible that imaging techniques may not readily detect less extensive ENE that has been identified by microscopy. The radiologic signs of ENE become prominent when the tumor has extensively penetrated the nodal capsule, resulting in a high specificity but low sensitivity. Less extensive ENE at its early stage is believed to manifest as irregular or distinct margins on radiologic images [14,15,40,41]. However, on StarVIBE sequences, this feature exhibits low sensitivity (SN 0.26) and low inter-rater reliability (ICC 0.56).

Our study has several limitations. Firstly, this study utilized a retrospective design and was conducted at a single academic institution, resulting in a limited number of patients and nodes. The availability of images for a comparative analysis between VIBE and StarVIBE was restricted to limited individuals. Consequently, no subgroup analysis was conducted regarding the tumor origin site and HPV infection, and it is essential to validate the results in alternative cohorts. A selection bias also exists as the patients in the cohort were optimally selected for neck dissection. Secondly, a direct comparison between the diagnostic performance of StarVIBE and CT is not possible due to the lack of neck CT scans or instances where CT was conducted at different institutions. Thirdly, the diagnosis of ENE relies solely on contrast-enhanced StarVIBE T1-weighted images, and the inclusion of functional MRI sequences needs to be investigated to further enhance diagnostic performance.

## 5. Conclusions

Our findings demonstrate that StarVIBE outperformed VIBE regarding improved overall image quality, motion, aliasing artifacts, SNR, and CNR of the abnormal lymph nodes in head and neck cancer patients. Among the various radiologic features on StarVIBE, matted nodes independently predicted ENE and served as valuable indicators for its diagnosis. Utilizing a combination of two features, namely matted nodes and invasive adjacent planes, StarVIBE had the potential to attain a high level of diagnostic accuracy for ENE before surgical intervention.

## Figures and Tables

**Table 1:** Baseline characteristics and demographics of the patient cohort.

Patient Characteristics	*n* = 80
Age, yrs (Mean ± SD)	61.1 ± 11.8
Gender	
Male	67 (84%)
Female	13 (16%)
Primary tumor site	
Oral cavity	24 (30%)
Oropharynx	1 (1%)
Larynx	16 (20%)
Hypopharynx	25 (31%)
Salivary gland	4 (5%)
pT	
T1	5 (6%)
T2	28 (35%)
T3	21 (26%)
T4	26 (33%)

**Table 2:** Comparison of image quality between StarVIBE and VIBE.

	StarVIBE	VIBE	*p*-Value	ICC (95%CI)
Overall image quality	5 (1)	3 (1)	<0.001 *	0.90 (0.82–0.94)
Motion artifacts	5 (0)	4 (1)	<0.001 *	0.90 (0.83–0.94)
Aliasing artifacts	5 (0)	4 (1)	<0.001 *	0.92 (0.87–0.96)
SNR	23.7 ± 9.8	15.1 ± 5.6	0.001 *	0.51 (0.26–0.69)
CNR	5.7 ± 3.2	3.0 ± 2.3	<0.001 *	0.79 (0.65–0.88)

* Statistically significant difference.

**Table 3:** Characteristics of cN+ patients with or without ENE.

	ENE− (*n* = 8)	ENE+ (*n* = 32)	*p*-Value
Age, yrs (Mean ± SD)	57.3 ± 16.0	62.1 ± 10.5	0.44
Gender			0.53
Male	6 (75%)	27 (84%)	
Female	2 (25%)	5 (16%)	
Primary tumor site			0.09
Oral cavity	6 (75%)	9 (28%)	
Larynx (Supraglottic)	0 (0%)	5 (16%)	
Hypopharynx	2 (25%)	15 (47%)	
Submandibular gland	0 (0%)	3 (9%)	
pT			0.28
T1	0	4 (13%)	
T2	2 (25%)	10 (31)	
T3	1 (13%)	9 (28%)	
T4	5 (63%)	9 (28%)	

**Table 4:** Sensitivity and specificity of MR feature for determination of pathologic ENE.

	Pathologic ENE				
	No	Yes	Sensitivity	Specificity	Accuracy
Irregular nodal margins					
No	14	40	0.26 (0.15–0.40)	0.74 (0.49–0.91)	0.38 (0.27–0.50)
Yes	5	14			
Invading adjacent planes					
No	19	25	0.54 (0.40–0.67)	1.00 (0.82–1.00)	0.66 (0.54–0.76)
Yes	0	29			
Nodal necrosis					
No	11	14	0.74 (0.60–0.85)	0.58 (0.34–0.80)	0.70 (0.58–0.80)
Yes	8	40			
Matted nodes					
No	17	15	0.72 (0.58–0.84)	0.89 (0.67–0.99)	0.77 (0.65–0.86)
Yes	2	39			
Size > 14.8 mm					
No	17	14	0.74 (0.60–0.85)	0.89 (0.67–0.99)	0.78 (0.67–0.87)
Yes	2	40			
Invading adjacent planes + matted nodes					
No	17	4	0.93 (0.82–0.98)	0.89 (0.67–0.99)	0.92 (0.83–0.97)
Yes	2	50			

**Table 5:** MR features on StarVIBE for determination of ENE compared to histopathology.

Variables	Univariate			Multivariate		
	OR	95% CI	*p*-Value	OR	95% CI	*p*-Value
Irregular nodal margins	0.98	0.30–3.22	0.98	0.31	0.02–5.00	0.41
Invading adjacent planes	Inf. *	N/A		Inf. *	N/A	
Nodal necrosis	3.93	1.31–11.75	0.01	5.45	0.52–56.70	0.16
Matted nodes	22.10	4.55–107.46	<0.001	57.52	3.48–951.95	0.005
Long axial diameter	1.36	1.14–1.62	0.001	1.11	0.81–1.52	0.51

* Infinite value, the specificity reached 1.00, and the OR was infinite.

## Data Availability

The datasets generated during the current study are available from the corresponding authors upon request.
